# Microvascular Tissue Engineering—A Review

**DOI:** 10.3390/biomedicines9060589

**Published:** 2021-05-21

**Authors:** Jernej Vajda, Marko Milojević, Uroš Maver, Boštjan Vihar

**Affiliations:** 1 Faculty of Medicine, Institute of Biomedical Sciences, University of Maribor, Taborska ulica 8, 2000 Maribor, Slovenia; jernej.vajda1@um.si (J.V.); marko.milojevic1@um.si (M.M.); 2 Department of Pharmacology, Faculty of Medicine, University of Maribor, Taborska ulica 8, 2000 Maribor, Slovenia; 3 IRNAS Ltd., Limbuška cesta 78b, 2000 Maribor, Slovenia

**Keywords:** regenerative medicine, tissue engineering, vascularization, biomaterials, microvascularization, coculture, gradients

## Abstract

Tissue engineering and regenerative medicine have come a long way in recent decades, but the lack of functioning vasculature is still a major obstacle preventing the development of thicker, physiologically relevant tissue constructs. A large part of this obstacle lies in the development of the vessels on a microscale—the microvasculature—that are crucial for oxygen and nutrient delivery. In this review, we present the state of the art in the field of microvascular tissue engineering and demonstrate the challenges for future research in various sections of the field. Finally, we illustrate the potential strategies for addressing some of those challenges.

## 1. Introduction

Blood vessels are often considered a uniform part of the circulatory system, maintaining a homeostatic environment in the tissues by supplying oxygen and nutrients and removing metabolic byproducts [1,2]. However, while the function of large vessels is conducting fluid through the body, microvasculature also enables the exchange of substances between the vascular lumen and the surrounding tissue [3]. Albeit there is no universal definition of microvasculature, it can best be described as a system of small diameter vessels (usually less than 100 µm) that exhibit a high surface-area-to-volume ratio and enable rapid exchange of fluid, solutes, and cells across the endothelial layer [3].

Today, vascularization stands as one of the most important challenges for creating stable, large tissues and organs in vitro. While tissue engineering and regenerative medicine have come a long way in recent decades [4,5,6], successful upscaling of tissue-engineered remains limited due to diffusion, which is insufficient for long-distance (>100–200 µm) delivery of oxygen and nutrients, as well as auxiliary functions such as waste removal and cellular communication [1,7,8]. Microvascularization is one of the unsolved challenges of vascular tissue engineering (VTE). Although it is very important for the future development of various branches of tissue engineering, this topic is currently only vaguely addressed in the available literature. Moreover, despite some interesting related research studies, to the best of our knowledge, there is no comprehensive review on this topic to date.

Currently, there are three main application areas for microvasculature engineering: therapeutic vascularization, creation of microphysiological system models in vitro, and vascularization of engineered tissues.

The aim of this review is to examine current approaches to microvascular tissue engineering to present the current state of the art of used materials, techniques, and cell sources, as well as their behavior while interacting with each other in native and simulated (engineered) tissues. Finally, the review concludes with the remaining challenges and future prospects of this important field, which intertwines to various degrees with all tissue engineering applications.

### 1.1. The Role of Microvascular Tissue in Tissue Engineering

**Figure 1:**
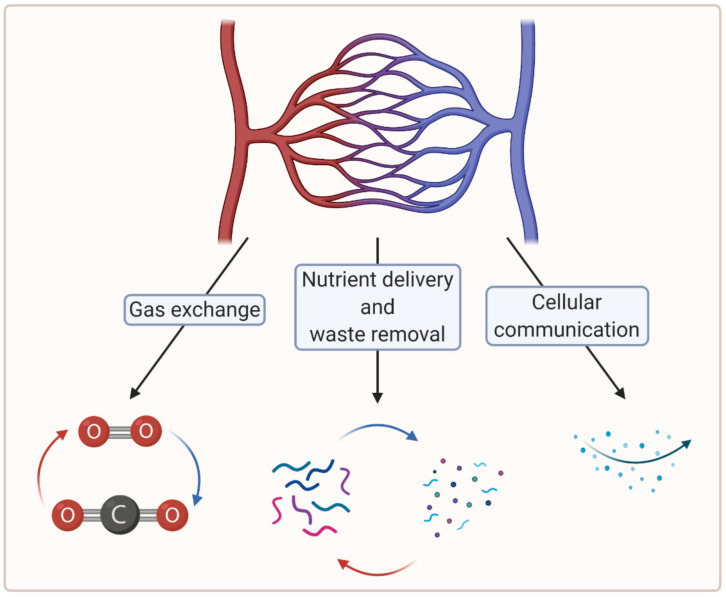
A schematic showing crucial functions of the microvasculature (created with BioRender.com; accessed on 6 April 2021).

To create large, viable engineered tissues, sufficient biochemical exchange beyond the diffusion limit (100–200 µm) is necessary, which in vertebrates is ensured by a cardiovascular system [7]. In this regard, microvascularization plays a crucial role in the distribution of oxygen and nutrients at the tissue level [8]. Moreover, microvascularization is key to the removal of waste material and plays a critical role in cellular communication [7,9]. Figure 1 summarizes the crucial functions of the microvasculature.

#### 1.1.1. Gas and Nutrient Exchange

Gas and nutrient exchange within tissues are vital for long-term tissue growth. Hypoxia is a state in which the local oxygen concentration is too low for tissues to survive long term. Some cell types are more resistant than others, but eventually, hypoxia leads to cell death, either by apoptosis or by necrosis [7,10]. It is important to distinguish the term hypoxia from hypoxemia, which refers to a low oxygen concentration in the blood, with 100% being 760 mm Hg, which is the standard atmospheric pressure [11,12]. Oxygen concentration gradually decreases from inhaled air to tissue level. Approximate values of the latter are 21.0% oxygen in the air at normal atmospheric pressure, 13.5% oxygen concentration in alveoli, 9.5% oxygen concentration in the arterial network, and 6.5% oxygen concentration in the venous network. In tissues, the physiological oxygen concentration is about 6.0% (ranging from 4.0% to 7.5%). Physiological hypoxia is the level at which normal hypoxic responses are elicited and range from 1.0 % to 5.0%, depending on the tissue. Pathological hypoxia that disrupts normal homeostasis is < 1.0% tissue oxygen concentration [12]. Camci-Unal et al. studied oxygen-releasing molecules incorporated into biomaterials to improve cell survival under hypoxic conditions. They showed that their use significantly decreased in vivo necrosis and lactate levels [13]. In this context, incorporating oxygen-releasing molecules within tissue-engineered constructs could eliminate the onset of hypoxia within the tissue from implantation to the formation of a functioning vasculature network [13]. What is more, oxygen-releasing biomaterials can potentially enhance vascularization and angiogenesis processes [13].

Tissues also require a continuous exchange of various substances other than gas: useful nutrients are transported into the tissues, while metabolic waste products must be removed to maintain cellular homeostasis [7,9].

Both gas and nutrient exchange can be maintained in thin constructs by diffusion alone. In thicker constructs, nutrients do not reach the core, leading to nutrient deficiency and also accumulation of waste products and eventually necrosis (in vitro and in vivo) [7,9]. In vivo, this is managed by the microvasculature, which is crucial for the long-term maintenance of adequate gas exchange at the microtissue level.

Therefore, to allow effective tissue growth in thicker constructs, microvasculature must also be established in artificial tissues.

#### 1.1.2. Cellular Communication via Endocrine and Paracrine Signaling

Cellular communication via endocrine signaling also depends on a functioning microvasculature. Organs with endocrine functions are especially densely permeated with a network of fenestrated capillaries. These allow the transit of various molecules through the endothelium into the vascular system, exploiting the latter for distribution to all parts of the organism [14]. A great example of this is pancreatic islets, which are surrounded by a microvascular network that is two to three times denser than the exocrine pancreatic tissue. Moreover, the endocrine cells in the islets are in close proximity to and polarized toward the vascular endothelial cells (ECs) of the islets and produce factors that promote the formation of fenestrae [14,15,16].

Cellular communication through paracrine signaling is also an important physiological aspect that is mediated in part by the microvasculature. Many cellular functions, such as cell differentiation, adhesion, and tissue repair, depend on paracrine signaling to respond correctly to their microenvironment [7,17].

Future studies of vascular and other tissue engineering types will need to consider these aspects to mimic native tissue effectively and allow for long-term in vitro tissue growth.

### 1.2. Anatomy and Histology of the Microvasculature

**Figure 2:**
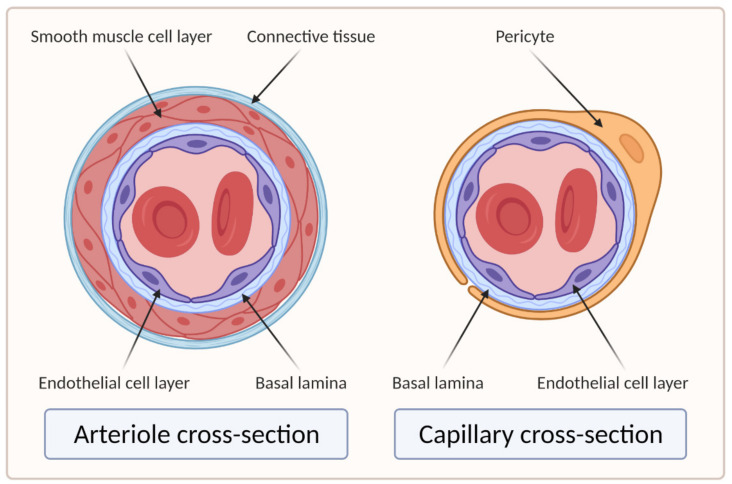
Cross sections of arteriole and capillary. On the left side of the image is the cross section of the arteriole showing four main layers: EC layer, basal lamina, smooth muscle cell layer, and connective tissue layer. The right side of the image shows the cross section of the capillary, which clearly lacks the smooth muscle cell and connective tissue layers compared to the arteriole (created with BioRender.com; accessed on 6 April 2021).

Larger vessels, such as arteries and veins, and arterioles and venules, have a thick, tough wall of connective tissue and many layers of smooth muscle cells, which are then lined by a single layer of ECs with a basal lamina between them. Capillaries, on the other hand, do not have connective tissue and smooth muscle cell layers but are composed of endothelial and basal lamina layers, with pericytes embedded in the basal lamina [18,19]. Figure 2 shows the difference in the cross section of an arteriole and a capillary.

## 2. Important Aspects of Microvascular Tissue Engineering

Microvascular tissue engineering has made great progress in recent years [1]. Significant success can be related to multidisciplinary approaches of materials science, additive manufacturing, topographic engineering, biomimicry, etc. [20]. An example of such an approach presents the spatiotemporal control over vascularization achieved by topographic engineering and controlled release of proangiogenic factors [20,21]. See Section 2.4. Gradients of Various Cues for further information.

Although many fundamental aspects of vascular tissue engineering apply to both large blood vessels and capillaries, fabrication methods for one vessel type are not necessarily applicable for the other. This is due to several profound differences between large vessel modeling and microvasculature modeling: vessel diameter (centimeters and millimeters vs. micrometers), number of vessels (individual hollow tubes vs. vascular network), the role of blood rheology (Newtonian vs. corpuscular fluid models), and characteristic Reynolds and Womersley numbers (relevant vs. very low) [22,23]. The fabrication methods are discussed in Section 3. Approaches to Microvascular Tissue Engineering.

Moreover, a single VTE approach is unlikely to be universally applicable; rather, it needs to be adapted to the target tissue. Tissue-specific cues play a crucial role in vascularization processes. Therefore, success is more likely using tissue-dependent development [1].

### 2.1. Choice of Materials

There are many different types of materials that can be used in vascular tissue engineering. Hydrogels, most commonly defined as systems of three-dimensional (3D), physically or chemically bound polymer networks that entrap water in the intermolecular space [24], are the most commonly used form of materials for this purpose, mostlyis because of their inherent property of structurally and biochemically mimicking the extracellular matrix (ECM). Their physical and chemical properties can be optimized by additives (e.g., nanofibrillated cellulose (NFC)) [25] to achieve optimal material properties for a specific application; additionally, different types can also be combined to form hybrid formulations with the same aim [9,26]. The ideal biomaterial should enable the formation of new blood vessels without toxic effects, induce the growth of vessels similar to native ones, and provide adequate mechanical support to the growing tissue. For example, scaffold stiffness is one of the most important tissue engineering parameters, namely, ECM stiffness has been shown to influence cell behavior, including adhesion, proliferation, migration, differentiation, signaling, and apoptosis [27,28,29]. By adjusting the concentration of, for example, alginate and carboxymethyl cellulose (CMC) from 0.1 to 8%, hydrogels with a varying stiffness from 0.1 kPa to 90 kPa can be produced [30,31], which is well within the range of soft tissues, such as striated muscles, or skin [32,33]. One of the most important parameters in the search for ideal materials for engineering specific tissues is their biodegradability. The latter should match the rate at which cells can replace the artificial ECM. Moreover, the degradation products should not negatively impact the developing tissue [2,34]. In addition to determining cellular behavior, the materials also influence the feasibility and strategies of fabrication [2].

Ink and bioink (ink containing live cells) components are typically divided into the following two categories: naturally derived and synthetic materials [9,35]. A number of naturally derived materials can be used for this purpose, which can be broadly categorized as either polysaccharides or proteins [9]. They are derived from a biological source, either animals (mammalian or nonmammalian), plants, or algae [2,9]. Naturally derived materials, especially proteins, can vary from batch to batch, increasing the variability of experimental results. Nevertheless, they exhibit superior biocompatibility and other characteristics that stimulate tissue development, such as proangiogenicity [9]. In addition to using purchased, defined materials, ECM materials can also be sourced from cells cultured in vitro [22]. Many cell types, especially fibroblasts, can deposit different ECM materials such as collagen, elastin, and fibronectin. The main advantage of this method is that this ECM has a native composition and properties that can induce physiologically relevant cell behavior [22,36,37].

Thus, the use of naturally derived source materials or even fully decellularized ECMs sets the standard for tissue engineering applications [38]. The targeted standard are artificially constructed ECM substitutes that allow more precise control and reproducibility. Some key input materials are discussed below.

Collagen is a commonly used material for microvessel engineering [1,9,22]. Type I collagen-based biomaterials have been shown to provide a suitable environment for angiogenesis. Therefore, it can stimulate the binding endothelial cell-surface integrins α_1_β_1_ and α_2_β_2_ via the GFPGER amino acid sequence of the collagen fibril. Moreover, ECs can degrade and invade the collagen matrix via metalloproteinases (MMPs) to establish vascular networks. This is mediated by collagen I-integrin interaction [1,9]. The macroscopic mechanical properties of the ECM and the behavior of the embedded cells depend on the microscopic orientation and fibrillar thickness of the collagen gel. Various techniques, such as electrospinning, stretching, and microfluidics, have been explored to control these properties [1,9,22].

Fibrin, one of the main components of the blood-clotting cascade, has also been used for microvascular tissue engineering due to its intrinsic angiogenic properties [22]. It is polymerized using fibrinogen and thrombin solutions and has been shown to promote cell migration, proliferation, and matrix synthesis [39] and facilitate successful vasculogenesis [40,41,42]. Cui et al. studied a bioink composed of human microvascular endothelial cells (HMVECs) and fibrin, for microvasculature construction. They precisely fabricated micron-sized fibrin channels using a drop-on-demand polymerization. Using this approach, they produced well aligned and straight fibrin fiber structures appropriate for cell seeding and microvasculature fabrication [39]. However, fibrin-only inks are generally poorly printable and have poor mechanical stability [9].

Another commonly used example of protein-based hydrogels is Matrigel^®^, a tradename for an ECM mixture that contains many factors, including laminin, nidogen, collagen IV, and heparan sulfate proteoglycans (perlecan). It is secreted by mouse tumor cells and resembles the complex basement membrane environment found in many normal tissues [43,44]. With its excellent proangiogenic properties, Matrigel^®^ has been designated as the standard substrate material in EC tube formation assay and in vivo angiogenesis tests. It can also be used as a supplement to other materials while retaining its proangiogenic effect. This was demonstrated in 2016 in a study by McCoy et al. in which lumenized angiogenic sprouting of human cerebral ECs was significantly improved when collagen hydrogel was supplemented with 2% (*v*/*v*) Matrigel^®^ [1,45]. Schumann et al. showed that preincubating mesenchymal stem cells (MSCs) in Matrigel^®^ presents a promising approach to develop rapid microvascular growth in tissue engineering constructs since the microvascular capillary-like structures developed exceptionally fast [46].

Gelatin methacryloyl (GelMA) is a semisynthetic hydrogel. It is based on a naturally derived material—gelatin—and contains methacrylate and methacrylamide groups. The mechanical properties of the gel can be fine-tuned by adjusting the degree of methacrylation. The bioactivity and tunability, as well as great mechanical stability of GelMA, make it an excellent candidate for direct bioprinting of microvasculature [9,47]. Chen et al. evaluated the use of GelMA in microvascular tissue engineering in a study in which they cocultured endothelial colony-forming cells (ECFCs) and MSCs and showed that ECFCs assembled into capillary-like networks [47]. However, the lack of MSCs in the culture and increased methacrylation degree negatively impacted the generation of capillary-like networks [47].

Alginate, a hydrophilic linear polysaccharide, as well as gelatin, chitosan, dextran, agarose, and hyaluronic acid also showed to have proangiogenic properties when modified with different functional groups [2,22,48]. For example, when an unmodified hyaluronic acid hydrogel was used, less successful vascularization was achieved, compared to a fibronectin-supplemented hyaluronic acid-based hydrogel [22,49]. Similarly, using hybrid alginate–chitosan microcapsule scaffolds for providing support and guiding alignment of human umbilical vein endothelial cells (HUVECs) resulted in vascular-like network formation [48].

A variety of other materials have been studied for microvascular tissue engineering. Due to their limited success for this purpose, the review does not include more detail in this regard. Some of these are polyethylene glycol (PEG) and propylene glycol diacetate (PGDA), polyglycolic acid (PGA), polycaprolactone (PCL), poly-L-lactic acid (PLLA), and elastin [1,9,22].

### 2.2. Choice of Cell Source

ECs are the main cell type for microvascular tissue engineering. They make up the inner lining of blood vessels and display inherent angiogenic behavior. Different types of ECs are commonly used in vascular tissue engineering. Among them, HUVECs, HMVECs, and induced pluripotent stem cell-derived endothelial cells (iPSC–ECs) are most commonly used. Vascular endothelial cells exhibit broad phenotypic heterogeneity, which is related to the tissue of their origin [50,51] and is a consequence of various (patho)physiological factors such as chemical (e.g., growth factors, hormones, cytokines) and mechanical cues [52]. For example, HMVECs can be categorized into adipose-tissue-derived, liver-tissue-derived, cardiac-tissue-derived, lung-tissue-derived, and dermal-tissue-derived subtypes. Although derived from different tissues, these subtypes of ECs share common markers, such as vWF and CD31 [1,9]. Evidence suggests that most of the phenotypic variability of ECs can be explained by environmental factors. Though epigenetic factors also seem to play a role to some extent, their impact is likely diluted through prolonged culturing [50]. Since the in vivo microenvironment remains difficult to be recapitulated to the full extent in in vitro settings, the morphology and function of ECs can differ significantly between the in vitro and in vivo studies [53]. The apparent plasticity of cells presents an opportunity for novel in vitro models. The use of a few cell types and appropriate environmental cues may cause the appropriate phenotype expression, as Nolan et al. showed that the cells’ microenvironment phenotypically and functionally “educates” ECs [54].

Of all the EC types, HUVECs have been the most studied. This is also the reason why the vast majority of microvascular models use HUVECs as the main cell type. However, since ECs are heterogeneous throughout the body, both in terms of physiology and functionality, the use of specific endothelial subtypes may be more suitable for specific tissue engineering applications [17,55].

#### Coculture

**Figure 3:**
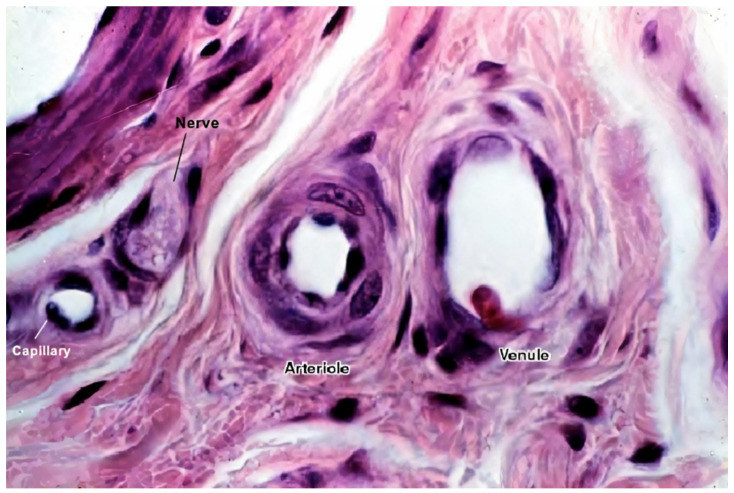
A histological image of different vessel types’ cross sections. The image shows the difference between different vessel walls—single layer wall of the capillary with only endothelial cells and occasional pericytes (not seen in this image), and multiple layer walls of arterioles and venules, which have many layers of muscle cells lining the endothelium [58]. For better visualization, the typical vessel diameters are as follows: arterioles (smallest, precapillary arteries), <100 μm; capillaries, 5–40 μm; venules (smallest, postcapillary veins), 10–200 μm [59]. Reprinted (adapted) from Creative Commons Attribution License CC BY-SA 4.0.

ECs alone are not sufficient for long-term vascular tissue culture—several additional supporting cell types are required that play a role in microvascular tissue engineering (see Figure 2 and Figure 3) [1], which include the following:

1. Mural cells line the endothelium. These include vessel-associated cell types, such as pericytes and vascular smooth muscle cells (vSMCs). Vascular smooth muscle cells are found predominantly on larger vessels, including arterioles and venules. Pericytes, on the other hand, are also found in capillaries. They provide mechanical support to ECs and manage the diameter of vessels and, according to recent studies, also regulate the permeability of the vessels [1,9]. Furthermore, they support angiogenic EC migration via MMP secretion, regulate endothelial permeability, and contribute to basement membrane formation [1,56,57];
2. MSCs secrete growth factors and thus promote blood vessel formation through angiogenesis. They are also the progenitor cells that can differentiate into both ECs and vSMCs (vSMCs are not directly associated with microvasculature as they only appear in larger diameter vessels) [9];
3. Fibroblasts also secrete many proangiogenic growth factors. Their main function is to secrete ECM proteins to reinforce the mechanical structure and promote the vascular network and lumen formation [9].

Figure 3 shows a histological image of different vessel types. See Figure 2 for a schematic cross-sectional comparison.

Cell–cell interactions play a major role in regulating vascularization development and coculture of different cell types. For example, a combination of ECs with fibroblasts has been shown to enhance angiogenesis in vitro [2].

When HUVECs were cultured alone, tube-like structures formed but quickly started to regress. In contrast, when HUVECs and MSCs were cocultured, MSCs were shown to migrate toward HUVECs and supported the formation and maturation of vascular networks [2,60]. Additionally, higher levels of endothelialization were observed in similar studies [61]. Chen et al. used ECs in coculture with hepatocytes. They showed that HUVECs started to form 3D capillary-like structures, whereas no such formation was observed when HUVECs were cultured alone [2,62].

Darland and D’Amore reported that when they cocultured HUVECs with fibroblasts or other stromal cells, capillary-like structures self-assembled without the addition of exogenous factors. They also showed that EC sprouting was robust in the presence of cocultured fibroblasts and intercellular lumens formed within 4–5 days. However, in the absence of fibroblasts, no vessels formed, and most cells died after 4–5 days [2,63]. Stromal cells, besides providing support for growth, were also found to wrap around ECs and take on a pericyte-like behavior [2,28,64]. Many studies have also shown that fibroblasts secrete soluble angiogenic growth factors such as vascular endothelial growth factor (VEGF), transforming growth factor-beta (TGF-β), and platelet-derived growth factor (PDGF), to name a few [63,65,66,67,68]. However, Marsano et al. studied VEGF overexpression in cardiac tissue patches and found that the therapeutic window of VEGF does not depend on the total VEGF in the tissue but rather on its concentration in the microenvironment around each producing cell since VEGF remains tightly bound to ECM [69,70,71]. They showed that localized high VEGF expression is sufficient to cause the formation of angiomas (vascular tumors). To prevent such processes, they controlled the distribution of VEGF by delivering monoclonal populations of transduced myoblasts, in which every cell produced the same amount of VEGF. This way, stable and functional angiogenesis was induced over a wide range of VEGF expression levels [69].

An important parameter in the preparation of cocultures is the ratio of ECs to tissue-specific cells, such as MSCs. The use of too many ECs in relation to tissue-specific cells decreases neovascularization of the graft, according to researchers [7,72]. To date, no ratio has been accepted as optimal as it depends on tissue type and graft size [7].

### 2.3. Environmental Cues That Control Angiogenesis

There are different environmental cues that stimulate angiogenesis. We divide them into two main groups: biochemical cues and biophysical cues.

#### 2.3.1. Biochemical Cues

**Figure 4:**
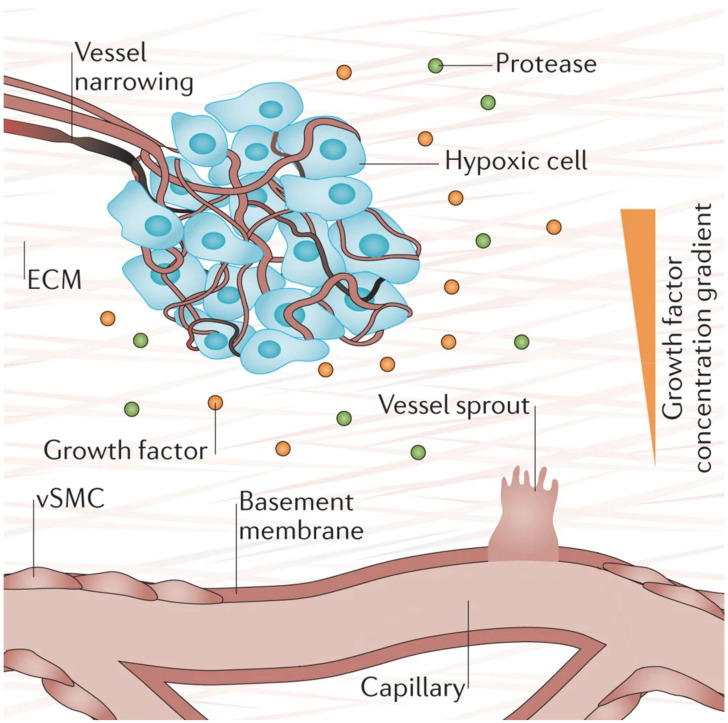
When there is insufficient oxygen supply to the cells (hypoxia), the cells begin to release growth factors that form gradients within the tissue that initiates new vessel sprouting. Reprinted and adapted with permission from Briquez, P.S. et al (2016) [76]. Copyright (2016) Springer Nature.

Hypoxia (low tissue oxygen level) is a crucial biochemical cue for vasculogenesis and angiogenesis, which promotes the sprouting of ECs toward the oxygen-deprived tissue [1,73]. It induces transcriptional responses in ECs that regulate proliferation, ECM degradation, pericyte recruitment, and sprouting [1]. Researchers have shown that these responses occur through the hypoxia-inducible factor (HIF)-dependent increase of VEGF transcription [73,74,75]. Figure 4 below depicts the process of hypoxia-induced vessel sprouting. For a more detailed illustration of this phenomenon, see the article by Briquez et al. [76].

VEGF, the main endothelial growth factor, and other angiogenic growth factors, such as fibroblast growth factors (FGF, released by fibroblasts) and angiopoietin 1 (Ang-1, released by smooth muscle cells), are the major stimulating factors for the development of new blood vessels [3,77,78]. Different combinations of growth factors have been shown to influence tissue development by affecting the number of vascular branches, branch length, diameter, and vascularized area [55]. It has also been shown that the addition of these factors to a 3D matrix (e.g., 3D-printed scaffold) enhances the formation of capillary-like tubular structures by inducing vasculogenesis and angiogenesis [2,79,80]. Ang-1 is required for the correct organization and maturation of newly formed vessels and promotes quiescence and structural integrity of adult vasculature [81]. Song et al. designed an experiment in which they cultured HUVECs in two parallel microfluidic channels. The addition of VEGF to the collagenous matrix resulted in HUVECs abandoning the preexisting vessel walls and sprouting into the 3D matrix, leading to endothelial sprout anastomosis. In contrast, without the addition of VEGF, the same experiment showed no anastomoses [2,80]. Growth factor gradients showed similar results, with ECs migrating toward higher VEGF concentrations [2,82].

A viable alternative to growth factor protein therapy is gene therapy, which potentially offers a more sustained presence of the desired protein in the engineered tissue [83]. Various clinical studies evaluated gene therapy with VEGF and other growth factors, such as FGF and HIF-1α. Some studies showed that such an approach resulted in a significant symptomatic improvement as well as angiographic evidence of enhanced collateral vasculature development; however, further studies are needed in order to establish different parameters of the technique, such as optimal duration and level of gene expression in angiogenic therapies [83,84,85,86].

Another way of stimulating angiogenesis is using cell-based therapy. It is conducted by injecting the target tissue with a cell line that would stimulate neovascularization. In 1997, Asahara et al. described putative endothelial progenitor cells (EPCs) that were thought to mobilize from the bone marrow to participate in neovascularization at sites of ischemia [87,88]. Since then, preclinical and clinical studies were undertaken in different animal models that evaluated the therapeutic angiogenic potential of bone marrow-derived cells (BMCs) thought to contain EPCs. They reported that the therapy functionally contributed to vascular regeneration in many different contexts, such as wound healing, graft reendothelialization, hindlimb ischemia, and myocardial infarction [88]. Besides EPCs, an ever-increasing number of other cell type candidates have been proposed, including, but not limited to, MSCs, adipose-derived stem cells (ADSCs), and embryonic stem cells (ESCs).

#### 2.3.2. Biophysical Properties

##### Static Properties

The biophysical properties of scaffolds play an important role in vascular tissue engineering because they influence cellular behavior in vitro. The most important biophysical properties include scaffold stiffness and pore size, as well as shear stress and internal architecture (alignment). The stability of the vessels is positively correlated with scaffold stiffness [2]. Research showed that stiff scaffolds with intermediate pore sizes (35–100 µm), and approximately 50% porosity yield the best results [2,73,89]. Larger pore sizes are not ideal since cells begin to migrate out of the channels into the scaffold. Smaller pore sizes, on the other hand, make the vessels unstable [2,73]. A minimum overall porosity of 50% and a pore size of 35–100 µm are considered optimal for blood vessel formation [73,89]. Surface topography and other physical properties can also affect cell behavior. For example, changes in wettability and electric charges can affect cell adhesion to a biomaterial surface. However, the exact biological mechanism of action is still unknown [73,90].

Although all these parameters have been proven to affect cell behavior, all previous studies have used a specific biomaterial and focused on a single biophysical parameter. A systematic study needs to be conducted to untangle the interdependency of different parameters on different materials [73].

Based on existing knowledge, gradients of biophysical and biochemical properties present an efficient way to study multiple parameters simultaneously. More on this follows in Section 2.4. Gradients of Various Cues.

##### Dynamic Properties

Fluid shear stress, the tangential component of the hemodynamic force, directly related to the flow rate, is among other chemical and physical factors that regulate EC development and migration [91]. It has been found to influence the development of EC monolayer; for example, Koo et al. showed that dynamic flow, applied to EC-seeded poly-(L-lactic acid) scaffold, enhanced EC migration [73,91]. Ueda et al. devised a study to determine the effect of shear stress stimulus on 3D microvessel formation in vitro. As a cell type, they used HMVECs from bovine pulmonary tissue and seeded them onto collagen scaffolds with incorporated basic FGF. After forming a microvascular network, the model was placed in a flow chamber, where laminar shear stress of 0.3 Pa was applied to the surfaces of the cells for 48 h. Increased microvascular network formation was detectable after around 10 h in the flow chamber. After 48 h, both the perfused network and the control network were evaluated. The results showed that the length of the perfused network was 6.17 (±0.59) times longer than at the initial state, while the length of the control network was only 3.30 (±0.41) times longer than at the initial state. The number of endpoints increased in the perfused network but not in a control network [92]. These results show that applied shear stress promotes the growth and development of the microvascular network. On the other hand, Song and Munn used a microfluidic perfusion device and reported reduced VEGF-induced sprouting under physiological shear stress [73,93]. Although most of the studies report positive effects of fluid shear stress, further studies are needed to obtain a clear picture (and quantification) of the effects of shear stress on endothelial development. The theoretical aspect and mathematical modeling of dynamic biophysical properties are discussed in Section 4. Mathematical Modeling of Biophysical Properties.

What is more, the use of flow has proven to be a promising technique of cell seeding in 3D scaffolds since researchers found that cells were able to migrate into the scaffold with the help of a flow perfusion system. Koo et al. showed that cells in static condition were unable to invade the microfibrous scaffold in 24 h, while cells in flow condition cells could easily penetrate the scaffold [91].

Electrical stimulation is another important biophysical cue for angiogenesis. It has been shown that the application of electric fields (EFs) of small physiological magnitude both directly and indirectly stimulates angiogenesis [94]. Zhao et al. found that applied EFs stimulate VEGF production by endothelial cells in culture without the presence of any other cell types [95]. Moreover, they showed that electrical stimulation also directed the reorientation, elongation, and migration of endothelial cells. It is interesting to note that different EF strengths impacted each of these processes [95]. Chekanov et al. used chronic low-frequency electrical stimulation in rabbit hindlimb ischemia cases and found that this approach increased capillary density after only 2 to 4 days of electrical stimulation. The total capillary surface area increased by 30 %, and the numerical density of arterioles increased by 100% during the initial 4 days of therapy but returned to baseline after 7 days [96].

### 2.4. Gradients of Various Cues

Biochemical and biophysical stimuli usually occur concurrently and often interact. Thus, prospective possibilities arise in microsystems that can combine both types of stimuli for the controlled modulation of cellular responses [97,98,99].

**Figure 5:**
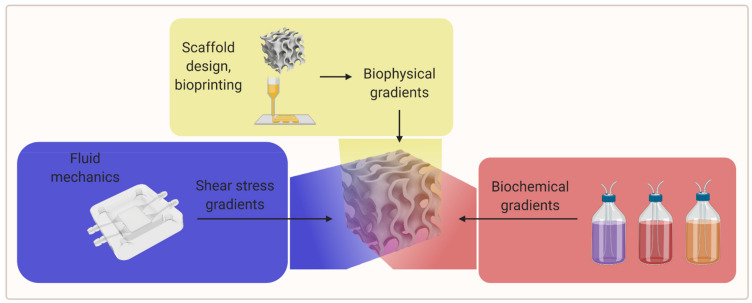
A visual overview of the gradient parameters for further research (created with BioRender.com; accessed on 20 May 2021).

An efficient way to study different material properties simultaneously while evaluating the influence of other experimental parameters could be the use of gradients of biophysical parameters (e.g., porosity, stiffness, topographical features) and biological as well as biochemical parameters (e.g., cell seeding density, scaffold crosslinking density, growth factor concentration, and growth factor isoform ratio) [2,9]. An overview of the gradient parameters is shown in Figure 5.

**Figure 6:**
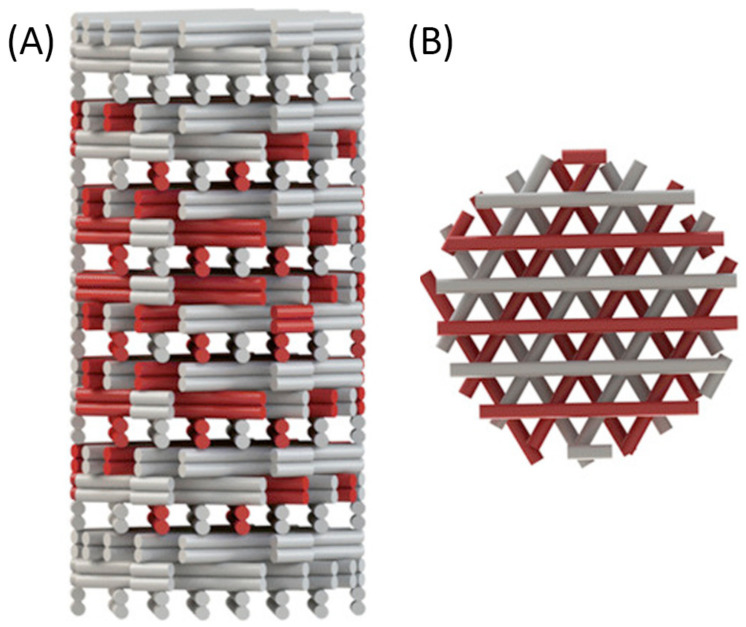
Gradient-like distribution of VEGF-loaded strands (red) within the scaffold: (**A**) side view of the scaffold showing different densities of VEGF-loaded strands in each layer; (**B**) top view of the same scaffold. Reprinted with permission from Bittner, S.M. et al. (2018) [21]. Copyright (2018) Elsevier.

There are studies of stiffness gradients in 3D hydrogel biomaterials, but there are few studies examining the cellular response to these gradients in a 3D environment. The study of cellular response has been mainly limited to 2D; therefore, further research is necessary to investigate stiffness gradients in 3D cultures [2]. Figure 6 is a schematic of a 3D-printed scaffold with a gradient-like distribution of VEGF within it. The coprinting of biochemical gradients in coordination with growth factors patterning is still an almost completely unexplored approach toward biomimetic scaffold fabrication that could be investigated in the future [21].

What is more, it has been shown that the magnitude and steepness of the growth factor gradient are aspects that play an important role in cellular response. A gradient steepness of 0.99 and 1.65 ng/(mL*µm) resulted in tubule-like structures that penetrated more than 200 µm into the scaffolds over, while a gradient steepness of 2.48 ng/(mL*µm) showed no evidence of tubule-like structures. It was also found that ECs were unable to migrate within gradient scaffolds containing VEGF concentrations above 600 ng/mL. Furthermore, it is important to distinguish between matrix-bound (immobilized) and soluble forms of growth factor since stable neovascularization is highly dependent on the established equilibrium between them [2].

## 3. Approaches to Microvascular Tissue Engineering

**Figure 7:**
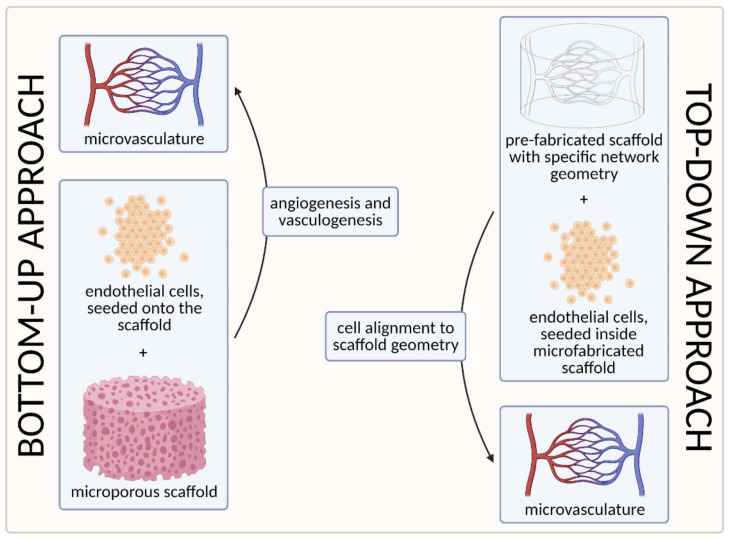
A visual comparison of bottom-up and top-down approaches in tissue engineering. In the bottom-up approach, ECs are seeded onto the microporous scaffold and the microvascular network is generated through vasculogenesis (including tubulogenesis) and angiogenesis. In the top-down approach, on the other hand, the scaffold with a specific network geometry is created using microfabricating techniques, with the aim of skipping the process of tubulogenesis [3] (created with BioRender.com; accessed on 6 April 2021).

To date, two main strategies have been used to develop the microvasculature in 3D—the “bottom-up” approach and the “top-down” approach [55,100]. The approaches are compared visually in Figure 7 below.

The first strategy, the so-called bottom-up approach, is to seed ECs in a 3D extracellular matrix, which would then spontaneously form vascular networks through vasculogenesis and subsequently sprout new vessels through angiogenesis, without any initial templating microstructures to guide the network development [55]. With this approach, vascular development can closely mimic in vivo conditions. However, the process cannot be (easily) controlled, and such 3D constructs are unlikely to be sufficiently perfused [3,100]. We discuss this approach in more detail in Section 3.1. Self-Organization Driven Bioengineering—the “Bottom-Up” Approach.

The other, the “top-down” approach, involves seeding ECs into separately microfabricated scaffolds with specific geometries and dimensions [100]. The underlying motivation is to skip the process of tubulogenesis or lumen formation that is required in the vasculogenic and angiogenic approaches [3]. Furthermore, the advantage of this approach also lies in the possibility to precisely control the vessel diameter and tightness of EC junctions by applying different flow parameters (e.g., shear stress) [100]. We discuss this approach in more detail in Section 3.2. Geometrically Defined Bioengineering—The “Top-Down” Approach.

Researchers have mostly focused on strategies based on chemical and biological cues when engineering vascular tissue. However, approaches involving physical properties such as shear stress, flow, scaffold stiffness, and geometry, are far less well studied [2].

### 3.1. Self-Organization Driven Bioengineering—The “Bottom-Up” Approach

**Figure 8:**
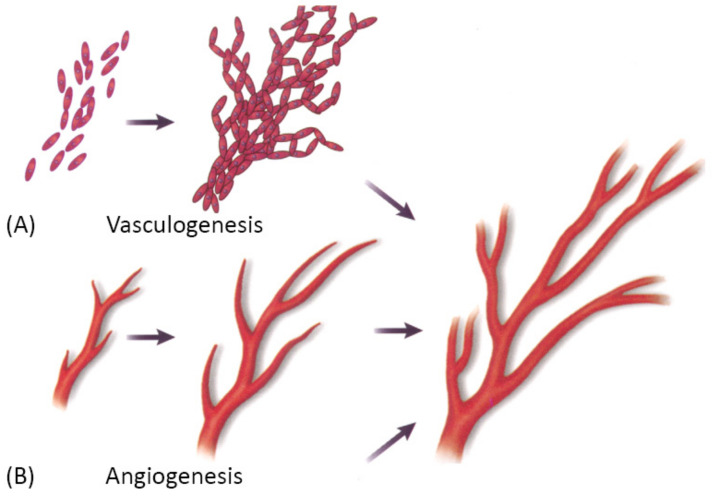
A schematic showing two main mechanisms of vascularization: (**A**) vasculogenesis, a process of spontaneous blood vessel formation from EPCs, and (**B**) angiogenesis, the formation of new blood vessels from preexisting ones through vascular sprouting. Reprinted with permission from Cleaver, O. et al. (1999) [102]. Copyright (1999) Elsevier.

Vascularization through a bottom-up approach is achieved by two different processes—vasculogenesis, which is the de novo assembly of endothelial progenitor cells that canalize into capillaries, and angiogenesis, the sprouting of new vessels from existing blood vessels. Since these processes are relatively slow, different growth factors are used to stimulate and accelerate vessel ingrowth [3,101]. Both processes are visually represented in Figure 8 below.

Vasculogenesis is mainly responsible for vascular growth in embryonic development. Embryonic vasculogenic formation of the vascular system begins when hemangioblasts organize into “blood islands” and then differentiate into hematopoietic stem cells and angioblasts. These then organize into interconnected solid cords and later undergo tubulogenesis to form a lumen. Further growth occurs through angiogenesis [3]. The principal question in microvascular tissue engineering by vasculogenesis is which cell population to use since differentiated ECs have been shown to be an inferior cell source for vasculogenesis than blood- and bone marrow-derived endothelial progenitors [3]. Cell types that have been used in various studies include differentiated ECs, EPCs, ADSCs, MSCs, endothelial outgrowth cells [3].

Angiogenesis is the main mechanism of vascular growth in some situations such as tissue healing or tumor growth [3]. It is a tightly regulated process, consisting of a series of well-defined steps: first, the ECM is degraded by MMPs; then, angiogenic factors are released to promote vascular sprouting, followed by elongation, branching, lumen formation, anastomosis, and finally, stabilization or regression [55]. As already mentioned, angiogenesis is dependent on stimulating factors, of which VEGF is the best known. However, many other factors also promote angiogenesis, such as FGF, Ang-1, etc. Moreover, VEGF itself is not a single growth factor but a family of many different growth factors that bind to specific receptors [3]. Since the field is still in the discovery phase, and many factors that promote angiogenesis in vivo are unknown, there has been only limited success in replicating the natural process [3].

“Bottom-up” fabrication methods do not require microstructures to guide ECs to form vessels in contrast to the “top-down” approach [55] discussed in the following section, Geometrically Defined Bioengineering—the “Top-Down” Approach. However, the main drawback of the method is the heterogeneity of physical parameters (diameter, length, geometry), which leads to an uncontrollable assembly of the vascular tree, its connection to the main circulatory system, and flow patterns [55].

The bottom-up fabrication approach has been previously used for tissue engineering, including microvascular tissue engineering. In 2013, Kusuma et al. utilized a synthetic hyaluronic acid-based hydrogel scaffold and seeded it with early ECs and early pericytes, differentiated from human induced pluripotent stem cells (iPSCs). After 3 days, complex vascular networks with patent luminal structures had developed, with ECs lining the lumens and pericytes encircling them. They implanted the vascularized scaffold into a murine model, where the vascular network anastomosed to host vessels, and the hydrogel scaffold was mostly degraded by week 2 [22,103]. Samuel et al. performed a similar study—they used the EPCs generated from human iPCSs, which formed a vascular network and remained stable for 280 days in a murine cranial model when co-implanted with murine fibroblasts in collagen [22,104]. Ueda et al. also used a collagen scaffold but incorporated the basic FGF instead and seeded it with bovine pulmonary tissue HMVECs. Using phase-contrast microscopy, confocal laser scanning microscopy, and electron microscopy, they observed that the cells invaded the collagen gel and reconstructed tubular structures with a clearly defined lumen consisting of multiple cells [92]. Modulevsky et al., on the other hand, took a native hypanthium tissue of apples, decellularized it, and implanted it into wild-type immunocompetent mice. Although there was an immune response in the first week following the implantation, the immune response gradually disappeared by 8 weeks postimplantation [105]. The scaffolds were resected, and the results showed that there had been active blood vessel formation within the scaffold, which indicates biocompatibility and even proangiogenic properties of the decellularized, yet otherwise unmodified plant cellulose [105,106]. It is important to note that vascularization occurred without any physical templating structures to guide the formation and without the need for biochemical functionalization of the cellulose scaffolds with proangiogenic factors [106].

### 3.2. Geometrically Defined Bioengineering—The “Top-Down” Approach

**Figure 9:**
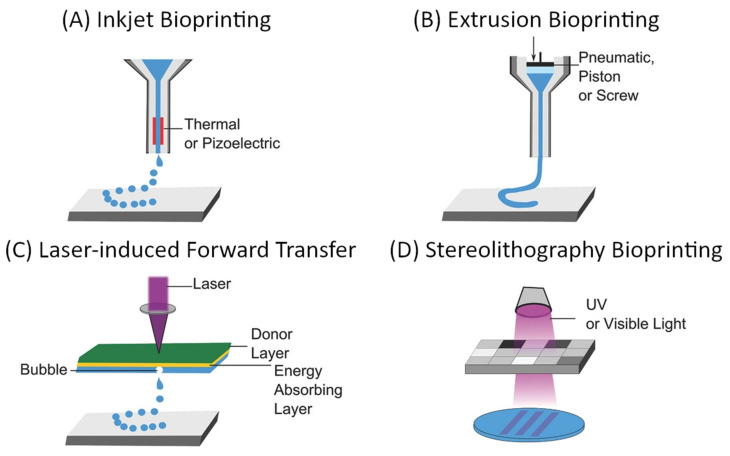
General overview of common approaches to additive manufacturing of scaffolds for tissue engineering: (**A**) inkjet-based bioprinting, (**B**) extrusion-based bioprinting, (**C**) laser-induced forward transfer, and (**D**) Stereolithography bioprinting [108,110]. Reprinted (adapted) from Creative Commons Attribution License CC BY 4.0.

The “top-down” approach to microvascular tissue engineering describes the process of direct, mechanical fabrication of scaffolds containing predetermined vessel pathways before cells are introduced [3,55]. Many approaches to directed patterning are possible and have been demonstrated as suitable for tissue engineering [5,6,107,108,109]. However, computer-aided design and manufacturing (CAD/CAM) approaches such as subtractive (e.g., soft lithography, laser ablation) or additive manufacturing (also 3D printing as a broad term for stereolithography, extrusion-based 3D printing, droplet-based 3D printing, etc.), and especially the latter have gained dominance in the biofabrication and tissue engineering landscape [55]. For microvascular tissue engineering, these fabrication methods demonstrate critical advantages since they allow precise control over vessel size and geometry, which are necessary to establish predictable flow patterns [55]. The most commonly used approaches to 3D bioprinting are summarized in Figure 9.

The main considerations for any 3D bioprinting approach include the geometric complexity and spatial resolution, which are possible using a certain technique, compatible materials, and biocompatibility of the used components as well as the process itself when cells are deployed during the fabrication (e.g., within bioinks). These aspects, as well as the (dis)advantages of the respective techniques, have been extensively discussed in some excellent reviews [5,6,107,108,109]. Techniques that need to be considered for the fabrication of vascular scaffolds are those particularly suitable for creating interconnected hollow channels, which are subsequently lined with an endothelial layer and connected to external perfusion [108]. In their 2020 review, Zhang and Khademhosseini outline four general approaches, which are convenient for the fabrication of such scaffolds: sacrificial printing, embedded printing, direct hollow fiber fabrication, and stereolithographic techniques [108]. The first three approaches rely on extrusion-based bioprinting, which is compatible with a wide range of materials (including natural and synthetic polymers and their hydrogels), enables rapid fabrication of relatively large structures, and is a cell-friendly process that allows the deployment of bioinks [5,6,107,108,109]. On the other hand, the main limitation of extrusion-based bioprinting is the spatial resolution, which is defined by the mechanical translation and the nozzle diameter, which typically lies in the 100 µm range [5,6,107,108,109]. Through the use of multiple printheads, and especially with the introduction of microfluidic and multicomponent extrusion systems, extrusion-based bioprinting allows spatial control over the chemical composition of scaffolds [111,112,113,114].

Using a 3D hydrogel scaffold with patterned hollow channels, Kolesky et al. showed preservation of EC phenotype and confluence for 6 weeks of active perfusion with an endothelium-specific medium [1,115]. Miller et al. managed to create interconnected orthogonal vascular networks within cell-containing fibrin hydrogels using a sacrificial material. The injected ECs formed single and multicellular sprouts from patterned vasculature [1,116]. Similar results were obtained using adjacent microfluidic channels injected with HUVECs, which were later assembled into continuous capillaries that anastomosed with the adjacent channels, allowing perfusion of the vascular network in vitro [1,117,118].

#### 3.2.1. Sacrificial Bioprinting

In addition to direct deposition of the scaffold material, extrusion-based printing for vascular tissue engineering often uses sacrificial inks that are embedded into the target scaffolding material and subsequently removed. The sacrificial ink can be deposited to a plain surface prior to embedding [115,119,120,121,122] or directly into a support bath that remains in place after ink removal [123,124,125,126]. In addition to the general properties that apply to microextrusion, support bath printing extends the range of appropriate materials and possible geometries of the final scaffolds. Since the inks stabilize on contact with the support bath, they retain the deposited shape without having to hold their own weight. This allows the use of inks with lower viscosity and the fabrication of delicate structures that would collapse on their own (e.g., with overhangs or containing large empty spaces, etc.) [108,124,125]. However, the support bath itself must also meet certain requirements, namely, (1) it should have “self-healing” properties, and continuously fill the void created by a moving nozzle; (2) the nozzle should displace only the material in its direct vicinity without disturbing the bulk material; and (3) if the support bath needs to chemically stabilize the deposited filaments, it should not prevent consecutive filaments from merging together on contact. These requirements are typically controlled by the composition and granulation of the support bath [108,124,125,126].

#### 3.2.2. Coaxial Bioprinting

Coaxial (i.e., core–shell or hollow fiber) printing exploits simultaneous extrusion of two or more materials, typically a (bio)ink, and a cross-linking solution, through a coaxial nozzle, causing the ink to stabilize at the interface of the two materials [26,108,127,128,129]. When the (bio)ink is extruded through the outer nozzle compartment, hollow fibers can be produced in a single-step process. Depending on the nozzle design, fiber diameter, wall thickness, etc. can be controlled [108]. In studies by Colosi et al. [113] and Costantini et al. [114], the coaxial printing process was also combined with microfluidic material assembly, which provides control over the longitudinal composition of the deposited material. Using coaxial printing to produce a continuous hollow fiber, larger, perfusable scaffolds can be manufactured [26,127,128]. The downside of this approach, however, is that the channels cannot be connected transversely, which is a major limitation in terms of the geometric complexity of the final structure.

#### 3.2.3. Stereolithography (SLA)

In contrast to extrusion, where printing resolution is defined by the nozzle and its positioning, the spatial resolution of photopolymerization is defined by the pixel size of a focused light beam and the precision of its positioning [6]. Typically, this allows the fabrication of significantly finer structures, especially when multiphoton effects are exploited. For example, Klar et al. demonstrated submicron resolution using multiphoton lithography [130] and that the approach can be used with biocompatible materials [131]. The curing of the material while surrounded by a liquid base provides stability to the scaffold and allows for the fabrication of highly complex geometries. The main limitation of SLA is the choice of compatible materials and an often harsh printing environment [6]. With the development of new materials and process optimization, it has become possible to 3D print complex structures using live cells [132]. However, to date, SLA remains inferior to extrusion-based in terms of material range, especially internal chemical gradients [111,112,113,114,132].

#### 3.2.4. Bioassembly

In addition to additive manufacturing using biological or biocompatible materials, bioassembly—the process of assembling pre-formed cellular building blocks [133]—combines bottom-up and top-down approaches to tissue engineering. The introduction of prevascularized building blocks into scaffolds can improve the vascularization of the final constructs [134]. Studies by Mineda et al. [135] and Bhang et al. [135] have demonstrated accelerated vascularization and regeneration in vivo by the introduction of spheroids. The success and usefulness of bioassemblies depend on the functionality of the preformed building blocks, spatial precision of block deposition, and the merging of the blocks into a compound unit. Consequently, bioassemblies are limited by both technical aspects of the top-down approach and the biological aspects of the bottom-up approach.

## 4. Mathematical Modeling of Biophysical Properties

Great progress has been made in the mathematical and computer modeling of complex microvascular systems in the last decades [23]. The use of such an approach serves several different functions in research, such as identifying key elements, testing hypotheses and simulations, and optimizing experimental methods, thus contributing to different stages in the understanding of biological phenomena [136,137].

There are four main areas of microvascular tissue engineering where computer modeling can be applied. These are modeling the geometry of microvascular network and surrounding tissue, modeling fluid dynamics, modeling gas transport, and modeling microvessel wall regulation [23].

**Figure 10:**
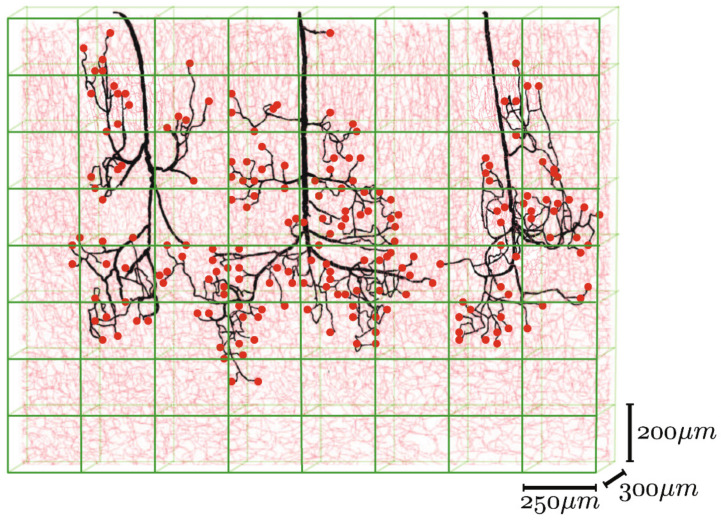
Mathematically generated capillary networks with different geometries [120]. Reprinted (adapted) from Creative Commons Attribution License CC BY 4.0.

Different mathematical algorithms are used to model the network-like structure of capillary beds [23]. Gould et al. and Safaeian et al. used Voronoi tessellation to generate the capillary beds [138,139]. Figure 10 shows various mathematically generated capillary networks. Ganesan et al. used a concentric circle mesh-like model to simulate capillaries in the murine retina [140]. Some studies have used sprouting angiogenesis algorithms to generate capillary networks embedded in tumor tissue [23]. Su et al. used statistical algorithms to explore how the transport of blood through the human cerebral microvasculature depends on the structural properties of the capillary bed [141]. Mathematical simulations also allow for more accurate design and prediction of optimal biomechanical properties, for example, pore size [73,142]. Since the surrounding tissue is an important part of microvascular tissue engineering, computer modeling of this tissue can also be of assistance [23].

Blood flow through the microvasculature differs considerably from the flow through larger vessels. The diameter of blood cells is comparable to vessel dimensions in the microvasculature, and cell–plasma interactions result in complex non-Newtonian dynamics [23]. For this reason, complex computer modeling could be an efficient way to determine approximate values for various flow parameters.

Oxygen and carbon dioxide are transported to and from the body tissues by circulation. The transport of gases from blood to tissues and vice versa is driven by partial pressure gradients. Most of the oxygen in the blood is bound to hemoglobin, a small fraction is dissolved in plasma, and similarly, oxygen in tissues is either dissolved or chemically bound to myoglobin. Therefore, the transport of oxygen involves convection, diffusion, and chemical reaction processes. Complex mathematical modeling is required to assess and successfully predict these processes [143]. Further details on the modeling of flow parameters and gas transport are beyond the scope of this review; we refer the reader to the study by Arciero et al. for more information [23].

Transmural pressure is an important parameter directly associated with vessel stability [2]. It can be calculated as the difference between the internal and external pressure of the microvessel and has two main components: active tension and passive tension. Since active tension is the result of smooth muscle cells’ contraction, which are not part of microvascularization, we will not discuss it in more detail here. Passive tension, on the other hand, is generated by the structural components of the vessel wall, which can be adjusted to optimize vessel stability [23].

## 5. Monitoring and Evaluation of Functionality

The evaluation of success in microvascular tissue engineering consists of two distinct modalities. The first is the evaluation of microvascular growth and development, and the other is the evaluation of tissue functionality [1,3].

Early studies relied on counting the number of histologically identifiable vessels per tissue volume to evaluate the success of microvascular growth and development. VEGF has been a global molecular marker of vasculogenesis and angiogenesis in recent decades. However, recent studies have used more refined approaches, such as staining for several specific endothelial markers, including, but not limited to, VE-cadherin (CD144), PECAM-1 (CD31), CD34, and nestin [1,3,144]. VE-cadherin is a strictly endothelium-specific adhesion molecule, located at the junctions between ECs. It controls EC contacts and blood vessel formation [145]. PECAM-1 is also highly expressed at junctions between ECs and functions as a mechanosensory that maintains the integrity of EC junctions [146]. Studies suggest that CD34 may function as an adhesion molecule on ECs [147]. Nestin is an intermediate filament protein and functions as a cytoskeletal structural component [1,148]. These approaches can be further improved with confocal microscopy, a method that enables us to assess the 3D distribution of markers and show the detailed geometry of the vascular network [3]. However, it is important to acknowledge the main limitation of the method—the penetration depth is limited to roughly 100 µm [149]. To achieve higher penetration depths, multiphoton microscopy with a penetration depth of up to 1 mm, or even optical coherence tomography, with a penetration depth of up to several millimeters, can be used [149].

However, microvascular tissue growth alone does not guarantee appropriate hierarchical vascular tree geometry and functionality, which is why researchers have recently started using other evaluation methods [1].

**Figure 11:**
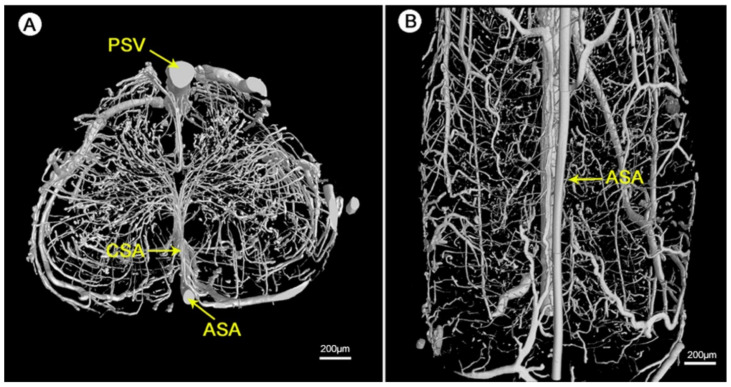
Visualization of the 3D microvasculature of the rat spinal cord by synchrotron radiation microcomputed tomography (SRµCT): (**A**) shows the transverse view and (**B**) shows the top view of the angioarchitecture [153]. Reprinted (adapted) from Creative Commons Attribution License CC BY 3.0.

The detailed geometry and functionality of the vascular tree can be assessed with various imaging techniques. The most commonly used techniques are magnetic resonance imaging (MRI) and nano-CT/micro-CT, complemented with an infusion of contrast agents or fluorescent lipids to evaluate the state of perfusability [1,3,150]. See Figure 11 for an illustration of micro-CT imaging. In addition, vascular permeability can be assessed by measuring the accumulation of a labeled solute in the surrounding tissue [3,151]. To evaluate in vivo vasculature, Demené et al. used ultrafast Doppler tomography (UFD-T) [1,152].

Since oxygenation of tissue cultures is an important regulator of cell function, especially in microvascular tissue engineering, measurement of oxygen levels can be crucial. Measurements of various biochemical and physical parameters such as local tissue O_2_ and CO_2_ levels, pH, concentrations of NO, H_2_O_2_, etc. are possible in real time with the use of microsensors without interrupting the experiment [154,155,156]. There are a number of manufacturers offering microsensors with tip diameters ranging from 10 µm for O_2_ and pH, 15 µm for NO, 100 µm for H_2_O_2_, and 250 µm for CO_2_ microsensors, according to the manufacturers’ websites (Unisense A/S, PreSens Precision Sensing GmbH, World Precision Instruments). The tip of the microsensor is inserted into the sample; for intricate work, micromanipulators can be used to achieve greater accuracy of the tip position [157].

To provide an efficient method for microvascular monitoring and evaluation, the above techniques should be used in combination to obtain additional, complementary information [1].

## 6. Conclusions and Outlook

**Figure 12:**
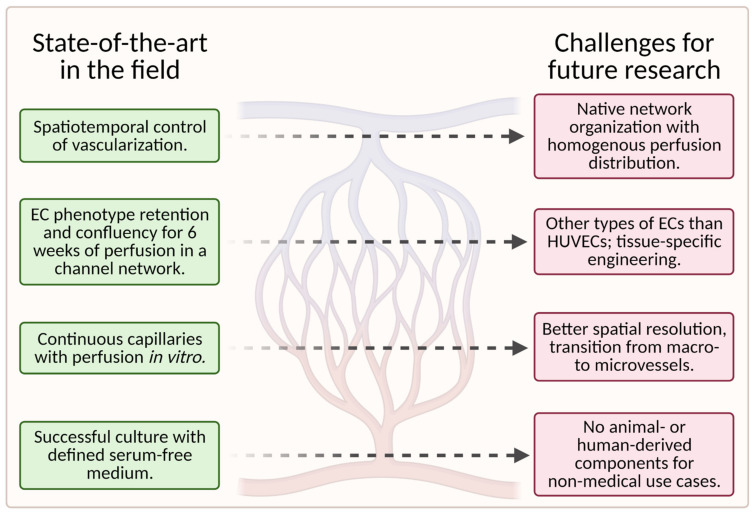
A schematic showing the current state of the art in the field (left column) and the associated challenges for future research (right column); (created with BioRender.com; accessed on 20 May 2021).

While tissue-engineering of vascular grafts has made significant progress in recent years, many challenges for future research remain. Figure 12 is a visual representation of the current state of the art in this field and the challenges (unsolved problems) for future research in this field.

To date, scaffolds fail to fully reproduce the native organization of the microvascular tree in tissue-engineered grafts. Instead, the capillary networks are “randomly” oriented and do not provide homogenous nutrient/waste exchange. More importantly, large hierarchically branched structures that transition from macrovessels to microvessels and finally capillaries have not yet been achieved [1].

The quality of the engineered vasculature cannot be maintained in vitro for prolonged periods, as reflected in endothelium homeostasis, perfusability, permeability, and contractility of the vessels [1].

Furthermore, the currently available fabrication methods utilized in the development of vascularized microfluidic models have limitations both in terms of channel size, particularly for the smallest diameters, and geometry. Even though advances in geometric complexity are being made with novel approaches such as stereolithography, it remains difficult to achieve geometric complexity while recapitulating capillary size [55].

Additionally, most successful studies used animal-derived materials for scaffold fabrication, which is unfavorable for nonmedical applications, such as cellular agriculture.

The identification of appropriate cell types (e.g., organotypic ECs or pluripotent stem cells) for specific applications requires further research, especially for the replication of specific tissues [55]. Most microvascular tissue engineering efforts have used HUVECs as the main EC subtype [55]. However, because HUVECs are derived from large veins, they may not fully recapitulate native microvessels such as arterioles and capillaries [9].

Moreover, tissue-specific cues play a pivotal role in vascularization processes; therefore, tissue-dependent development will be vital for success [1]. An efficient way to study various material properties and chemical parameters simultaneously could be the use of gradients—the topic is addressed in Section 2.4 Gradients of Various Cues.

The future looks bright, especially considering that both regenerative medicine and cellular agriculture are pushing for novel and more efficient solutions for artificial tissue vascularization. Current developments indicate that the simultaneous development of (1) advanced materials with dynamically adaptable mechanical, physicochemical, and structural properties; (2) more precise microadditive production methods to build more complex bioscaffolds; (3) the use of new cellular sources; as well as (4) optimization of cell culture conditions will lead to the construction of in vitro microvasculature that fully and representatively mimics the hierarchical structure and functionality of tissue vasculature in vivo.

## Data Availability

Not applicable.
